# A Small Price to Pay: National Narcissism Predicts Readiness to Sacrifice In-Group Members to Defend the In-Group’s Image

**DOI:** 10.1177/01461672221074790

**Published:** 2022-02-22

**Authors:** Bjarki Gronfeldt, Aleksandra Cislak, Anni Sternisko, Irem Eker, Aleksandra Cichocka

**Affiliations:** 1 University of Kent, Canterbury, UK; 2 SWPS University of Social Sciences and Humanities, Warszawa, Poland; 3 New York University, New York City, USA; 4 Mugla Sitki Kocman University, Muğla, Turkey

**Keywords:** COVID-19, collective narcissism, national identification, group reputation

## Abstract

Collective narcissism is a belief in one’s in-group greatness that is underappreciated by others. Across three studies conducted in the context of the coronavirus disease 2019 (COVID-19) pandemic, we found that collective narcissism, measured with respect to the national group, was related to support of policies that protect the national image at the expense of in-group members’ health. In Study 1, British national narcissism was related to opposing cooperation with the European Union (EU) on medical equipment. In Study 2, American national narcissism predicted opposition to COVID-19 testing to downplay the number of cases. In Study 3, American national narcissism was related to support for releasing an untested COVID-19 vaccine, to beat other countries to the punch. These relationships were mediated by concern about the country’s reputation. Our studies shed light on collective narcissism as a group-based ego-enhancement strategy in which a strong image of the group is prioritized over members’ well-being.

Concern for the welfare of one’s fellow citizens and showing the world that one’s nation is strong and independent do not always go hand in hand. Sometimes, especially in times of crisis, the two can be pitted against each other, resulting in a dilemma between protecting the people and conveying a positive image of the nation. This may be one factor explaining why governments have differed widely in their approaches to the coronavirus disease 2019 (COVID-19) pandemic. Although some have successfully contained the virus, others seemed more preoccupied with managing their country’s image. Anecdotally, countries with leaders holding exceptionalist views of their nation, such as the United States and the United Kingdom, had some of the worst early responses to this public health crisis (Lincoln, 2020). For example, former U.S. President Donald Trump called for testing to be slowed down, explicitly because more cases would make the United States look bad in comparison to other countries (Segers, 2020). Similarly, the United Kingdom declined to cooperate with the European Union (EU) on medical equipment, explicitly to underline the country’s departure from the bloc (BBC News, 2020). Such excessive focus on displaying a strong in-group image is a central concern in collective narcissism—a belief in the greatness of one’s in-group that is seemingly undervalued by others (Golec de Zavala et al., 2009). In this article, we utilize the context of the COVID-19 pandemic to examine whether collective narcissism in relation to one’s national in-group (which can be referred to as national narcissism) may be associated with a preference for promoting a strong image of the nation over protecting one’s own fellow citizens.

## Collective Narcissism as Superficial In-Group Love

Collective narcissism entails an excessive emotional investment in an unrealistic belief about the in-group’s greatness. Yet, this belief is defensive and linked to a conviction that others do not appreciate the group enough (Golec de Zavala et al., 2009). Collective narcissism parallels individual narcissism, but rather than capturing beliefs about the self, it captures beliefs about one’s social group. Individual and collective narcissism show only weak to moderate correlations (Golec de Zavala et al., 2019) and have different consequences for interpersonal and intergroup relations (Golec de Zavala et al., 2009). For example, those high in individual narcissism are aggressive toward individuals who threaten their personal ego, while those high in collective narcissism are aggressive toward members of other groups perceived to threaten or insult the in-group (Golec de Zavala, Cichocka, & Iskra-Golec, 2013; Golec de Zavala et al., 2016).

Research shows that those scoring high in collective narcissism are especially motivated to defend their in-group image when they are threatened by intergroup comparisons (Golec de Zavala, Cichocka, & Iskra-Golec, 2013). They tend to be excessively preoccupied with how others see them, and will engage in out-group derogation to maintain the in-group’s reputation (Cichocka, 2016). This is likely one of the reasons for the relationship between national narcissism and support for populist or nationalist leaders and movements that promote the narrative that their countries have been snubbed in international relations, such as Trump in the United States (Federico & Golec de Zavala, 2018) or Law and Justice in Poland (Marchlewska et al., 2018).

Even though collective narcissism might superficially look like strong commitment to the in-group, people high in collective narcissism are more concerned with how the in-group image reflects on themselves, than with the well-being of other in-group members. Those high in collective narcissism seem to compensate for their own frustrated individual needs by glorifying their in-group (Cichocka, 2016), indicating that collective narcissism serves as a group-based ego enhancement strategy (Cichocka & Cislak, 2020). For example, collective narcissism tends to increase when individuals feel that they have low personal control of their lives (Cichocka et al., 2018) or when their feelings of self-worth are threatened (Golec de Zavala et al., 2020). Overall, this suggests that collective narcissism does not develop out of genuine concern about the in-group. Instead, it emerges from a frustration at the individual level, which manifests as superficial in-group love (Marchlewska et al., 2020). This explains why people high in national narcissism are willing to leave their country if it benefits them financially (Marchlewska et al., 2020).

## Sacrificing the In-Group to Defend Its Image

Although various theories have attempted to explain *self*-sacrifice for the sake of the in-group (see Whitehouse, 2018), acceptance of in-group suffering has received limited attention. Kahn and colleagues (2017) found that perceiving the national in-group as a trans-generational entity (comprising of past, present, and future members), rather than consisting merely of contemporary group members, predicted willingness to endure in-group suffering. In other words, how we think about our in-group influences our acceptance of sacrificing fellow group members for the perceived benefit of the group at large.

A different process might accompany collective narcissism. It appears that those high in collective narcissism view their fellow in-group members as dispensable. For example, they tend to objectify their in-group members and use them as means to an end (Cichocka et al., 2021). This may be a result of the interplay between an instrumental view of the in-group (Cichocka, 2016) and an obsession with its image (Golec de Zavala et al., 2016). This toxic blend may lead those high in collective narcissism to view in-group members as an acceptable sacrifice for the maintenance of a desirable in-group image. The COVID-19 pandemic often pits in-group image concerns against the in-group’s health and wellbeing. For instance, there may be trade-offs between in-group members’ health and a perceived image of the group as strong and independent (see, for example, Cislak, Marchlewska, et al., 2021). In such situations, we expect those high in collective narcissism to view compatriots as acceptable collateral damage.

For those high in collective narcissism, low regard for in-group members and high concern about the in-group’s image can translate into support for policies that may have harmful implications for the in-group. For example, in Poland, national narcissism predicted anti-environmental attitudes, such as, support for the coal industry or for logging a unique, ancient forest (Cislak et al., 2018). The underlying motivation seemed to be to demonstrate that “our country” will make independent decisions and resist pressures from outsiders. In other words, collective narcissism seems to be linked to preference for policies that prioritize the in-group image in the short term over actions promoting health and well-being of in-group members in the long term (Cislak, Cichocka, et al., 2021). This can also manifest in behavior in one’s personal life. For example, recent research found collective narcissism to be related to short-sighted responses to the pandemic, such as hoarding food and supplies (Nowak et al., 2020).

Collective narcissism has also been associated with negative attitudes toward supranational or international organizations. For example, both British and Polish national narcissism were related to support for leaving the EU (Cislak et al., 2020; Golec de Zavala et al., 2017; Marchlewska et al., 2018). This may be due to a desire to assert independence or establish recognition of the in-group (Cichocka & Cislak, 2020). Those high in collective narcissism may also forgo help from outsiders. A study by Mashuri and colleagues (2020) demonstrated that national narcissism in Indonesia was related to refusing humanitarian aid from developed countries. The effect was driven by conspiracy beliefs about malignant intentions behind the offer as well as the perception that accepting humanitarian aid would damage the in-group’s reputation. This could be because groups that offer help are seen as having higher status than groups requesting or receiving help (Täuber & van Leeuwen, 2017). Indeed, receiving help from outsiders can be painful to some group members and even give rise to defensiveness (Bruneau & Saxe, 2012; Nadler & Halabi, 2006). National narcissism has also been associated with anti-science attitudes that would undermine the public health of in-group members, such as opposition to compulsory vaccinations (Cislak, Marchlewska, et al., 2021). Resource exploitation, opposition to humanitarian aid, and voluntary vaccinations may serve to maintain a positive or strong in-group image by asserting independence from external forces, such as powerful pharmaceutical companies, scientists, or malevolent foreign countries. In other words, these counterproductive responses aimed at upholding the country’s reputation in the eyes of the world may serve to manage the national image.

## Image Management in the COVID-19 Pandemic

Potential problematic consequences of natural exploitation or leaving international organizations might not materialize immediately. However, the COVID-19 pandemic is an imminent threat. Because those high in national narcissism have a strong desire to portray a positive in-group image, one may predict that they would support drastic measures to do so. However, having an exceptionalistic view about one’s nation may sabotage their effectiveness (Lincoln, 2020). Responses to the COVID-19 pandemic often created a dilemma: whether to support fellow citizens, or to reinforce the in-group image. As the primary concern of those high in national narcissism is an ideal image of the nation (Cichocka & Cislak, 2020), we predict that those high in national narcissism resolve this dilemma in favor of reinforcing the in-group image rather than prioritizing fellow citizens’ health. Furthermore, we predict that this relationship should be mediated by national reputation concerns due to the obsession of those high in national narcissism with what outsiders think of the in-group (Golec de Zavala et al., 2016).

## Overview

In this project, we examine the relationship between national narcissism and readiness to prioritize image management over in-group member’s welfare. We also examine a possible underlying process of this relationship: concern about the in-group’s reputation. We address three specific instances of when COVID-19 measures led to a dilemma between national image concerns and public health: (a) the United Kingdom’s cooperation with the EU on medical equipment early in the pandemic, (b) limiting COVID-19 testing in the United States, and (c) the debate on a premature release of a vaccine for COVID-19 in the United States. In these contexts, we directly ask participants whether they would accept harm to in-group members to protect the nation’s image.

Because national narcissism tends to be positively correlated with national identification (Golec de Zavala, Cichocka, & Bilewicz, 2013), we included national identification as covariate in all studies. We also adjusted for support for the country’s government in light of past work connecting national narcissism and support for Trump and Brexit (Golec de Zavala et al., 2017; Marchlewska et al., 2018). All studies were approved by the University of Kent Ethics Committee. Unless noted otherwise, patterns of results remained similar adjusting for demographics (age, gender, and ethnicity). Please refer to the Supplement for analyses including measures of ethnicity.

## Study 1: U.K. Cooperation With the EU on COVID-19

Early in the pandemic, the EU invited the United Kingdom to participate in the so-called “ventilator scheme,” in which the EU used the economic force of its single market to procure medical equipment that was in high demand (Stone, 2020). However, the U.K. government announced that it would procure medical equipment on its own (“‘Mix-up’ over EU Ventilator Scheme,” 2020). This decision was heavily criticized. Many argued that Prime Minister Boris Johnson prioritized his “Brexit ideology” over the welfare of his compatriots (Stone, 2020).

In Study 1, we utilized a real news story about the EU ventilator scheme (Stone, 2020). We hypothesized that national narcissism would be positively associated with support for the government’s decision to opt out of the scheme. To tap more directly into in-group sacrifice, we hypothesized that national narcissism would be positively associated with support for the decision to opt out even though it could hurt Britons. Moreover, we predicted that national narcissism would be positively associated with group reputation concern, and that group reputation concern would mediate national narcissism’s relationship with willingness to sacrifice in-group members. This study was not pre-registered.

### Method

#### Participants and procedure

Study 1 relied on a convenience sample of British people. Participants were recruited on several Facebook groups where politics are frequently discussed (i.e., pro Brexit and pro Remain/Rejoin platforms and several local community pages). In total, 298 participants completed an online survey (61.41% women, *M*_age_ = 54.25, *SD* = 13.65, age range 19–86). Most participants (92.95%) were White and held a university degree (66.45%). Because the topic was prominent in the news cycle in late March 2020, we restricted the sampling period to 3 days and aimed to recruit as many participants as possible (March 28–30, 2020). A G*power sensitivity analysis suggested that this sample size provides 80% power to detect a small or small-medium effect for a single regression coefficient (*f*^2^ = .03), assuming α = .05, two-tailed.^1^

The survey included measures of national narcissism, identification, politics, attitudes toward the EU, and demographic questions. Presentation order of variables was randomized, in that participants either first completed measures of national identification and national narcissism, or first saw a news story about the United Kingdom refusing to join the EU “ventilator scheme“ (see more detailed description below). Presentation order did not affect the pattern of results.

All data, codebooks, codes, materials, and pre-registrations are available at: https://osf.io/t6qrz/

#### Measures

In all studies, unless otherwise noted, participants were asked to indicate their agreement with survey items on a scale from 1 = *completely disagree* to 7 = *completely agree*.

#### National narcissism

National Narcissism was measured with the nine-item Collective Narcissism Scale (Golec de Zavala et al., 2009). Participants were asked to indicate how much they agreed with statements such as “Britain deserves special treatment” and “If Britain had a major say in the world, the world would be a much better place” (α = .90, *M* = 2.37, *SD* = 1.33).

#### National identification

National Identification was measured with the 12-item Social Identification Scale (Cameron, 2004). The scale measures centrality (e.g., “In general, being a British person is an important part of my self-image”), ties with other in-group members (e.g., “I feel strong ties with other British people”) and in-group effect (e.g., “Generally, I feel good when I think about myself as a British person”; α = .86, *M* = 3.94, *SD* = 1.18).

##### Support for opting out of the EU ventilator scheme

Participants read a passage, adapted from a real news story in *The Independent* (Stone, 2020). They learned that the United Kingdom had been invited to participate in a scheme initiated by the EU on using the purchasing power of the single market to procure in bulk ventilators and other much-needed medical equipment, guaranteeing lower prices and faster delivery. The U.K. government turned this offer down and decided to acquire the medical equipment independently. Participants were asked how much they agreed or disagreed with this decision (*M* = 2.11, *SD* = 1.98).

##### Sacrifice of in-group members

We measured participants’ willingness to sacrifice fellow Britons with one item: “Even if refusing to participate in the EU scheme ends up hurting British people, it would still have been the right decision” (*M* = 1.78, *SD* = 1.67).

##### Group reputation concern

We measured participants’ perception of their in-group’s reputation being threatened with one item: “The UK’s reputation in the world would have been damaged by participating in the EU scheme” (*M* = 1.83, *SD* = 1.67).

#### Political Ideology

Political Ideology was measured with a single item. Participants were asked to indicate where on a scale of 0 to 10, where 0 is *left-wing* and 10 is *right-wing*, they would place their political views (*M* = 3.69, *SD* = 2.15).

##### Satisfaction with Boris Johnson’s leadership

Participants were asked to indicate their satisfaction with Boris Johnson’s leadership on a scale from 0 = *very dissatisfied* to 10 = *very satisfied* (*M* = 2.67, *SD* = 3.56).

### Results

Zero-order correlations are presented in Table 1. To test our hypotheses, we conducted a series of multiple regression analyses (see Table 2).^2^ In all models, national narcissism and national identification were entered as predictors in Step 1. In Step 2, we also adjusted for political ideology and support for Johnson.

**Table 1.** Zero-Order Correlations Among Study Variables.

Variable	1	2	3	4	5	6
1. National narcissism						
2. National identification	.54***					
3. Opting out of EU scheme	.59***	.52***				
4. Sacrifice of in-group members	.59***	.35***	.60***			
5. Group reputation concern	.53***	.34***	.60***	.61***		
6. Right-wing ideology	.55***	.48***	.49***	.47***	.44***	
7. Satisfaction with Johnson	.62***	.59***	.71***	.56***	.51***	.56***

*Note.* EU = European Union.

* *p* < .05. **p < .01. ***p < .001.

**Table 2.** Regression Analysis of Support for Opting Out of the EU Scheme, Sacrifice of In-Group Members and Group Reputation Concern.

Predictor	DV: Opting out of EU scheme						DV: Sacrifice of In-group members						DV: Group Reputation Concern					
	Step 1			Step 2			Step 1			Step 2			Step 1			Step 2		
	*b*	95% CI	*β*	*b*	95% CI	β	*b*	95% CI	β	*b*	95% CI	β	*b*	95% CI	β	*b*	95% CI	β
National narcissism	0.65***	[0.49, 0.81]	.44	.30***	[0.14, 0.46]	.20	0.71***	[0.54, 0.85]	.57	0.48***	[0.33, 0.64]	.39	0.61***	[0.47, 0.76]	.49	0.39***	[0.23, 0.55]	.31
National identification	0.48***	[0.30, .66]	.29	.15	[−0.02, 0.32]	.09	0.06	[−0.10, 0.21]	.04	−0.15	[−0.31, 0.02]	−.11	0.10	[−0.06, 0.27]	.07	−0.09	[−0.26, 0.08]	−.06
Right-wing ideology				.05	[−0.04, 0.14]	.05				0.11*	[0.02, 0,20]	.14				0.12*	[0.02, 0.21]	.15
Satisfaction with Johnson				.28***	[0.22, 0.34]	.50				0.14***	[0.08, 0.20]	.30				0.13***	[0.07 0.19]	.28
*F* (*df*)	99.79 (2, 290)***			87.89 (4, 288)***			75.65 (2, 289)***			52.41 (4, 287)***			56.86 (2, 290)***			38.49 (4, 286)***		
*R* ^2^	.41			.55			.35			.42			.28			.35		

*Note.* DV = dependent variable; CI = confidence interval; EU = European Union.

* *p* < .05. **p < .01. ***p < .001.

#### Support for opting out of EU ventilator scheme

We first examined national narcissism and national identification as predictors of support for opting-out of the ventilator scheme (Step 1; Table 2). National narcissism (β = .44, *p* < .001) and national identification (β = .29, *p* < .001) were both significant, positive predictors of support for opting-out of the ventilator scheme. When we adjusted for right-wing ideology and satisfaction with Johnson in Step 2, national narcissism remained a significant predictor (β = .20, *p* < .001), but national identification was no longer significant (β = .09, *p* = .081). Furthermore, satisfaction with Johnson was significantly associated with support of opting-out (β = .50, *p* < .001), while the effect of right-wing ideology was not significant (β = .05, *p* = .300).^3^

#### Sacrifice of in-group members

We then tested a regression model with willingness to sacrifice in-group members as the dependent variable. In Step 1 (see Table 2), national narcissism emerged as a significant, positive predictor (β = .57, *p* < .001), while the effect of national identification was not significant (β = .04, *p* = .482). When we included the adjustment variables in Step 2, national narcissism remained a significant, positive predictor of willingness to sacrifice in-group members (β = .39, *p* < .001), while national identification was not a significant predictor (β = -11, p = .075).Furthermore, both right-wing ideology (*β* = .14, *p* = .017) and satisfaction with Johnson (β = .30, *p* < .001) were significant predictors of willingness to sacrifice in-group members.

#### Group reputation concern

We tested regression models with group reputation concern as the dependent variable. In Step 1, national narcissism emerged as a significant, positive predictor (β = .49, *p* < .001), while the effect of national identification was not significant (β = .07, *p* = .213). National narcissism remained significant in Step 2 (β = .31, *p* < .001). Both right-wing ideology (β = .15, *p* = .016) and satisfaction with Johnson (β = .28, *p* < .001) predicted group reputation concern in Step 2.

#### Group reputation concern as a mediator for opting out and sacrificing in-group members

**Figure 1.**
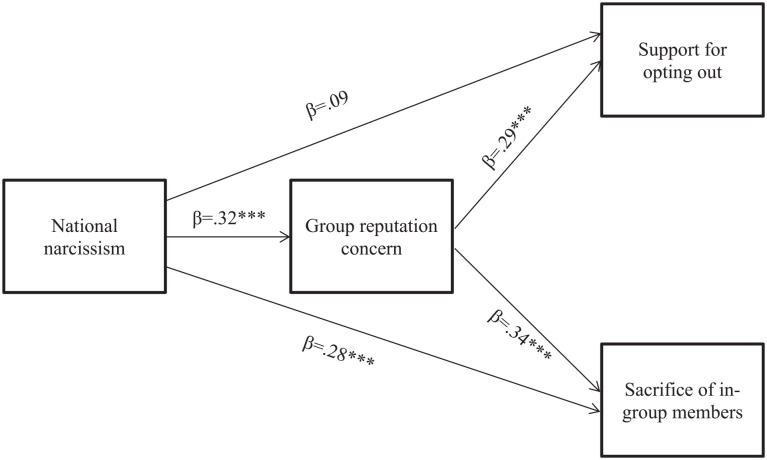
Group reputation concern as a mediator of the relationship between national narcissism and the dependent variables. *Note.* Entries are standardized coefficients. Covariates in the model are national identification, satisfaction with Johnson, and ideology. *p < .05. **p < .01. ***p < .001.

We then tested whether group reputation concern mediates the relationships between national narcissism and support for opting out of the EU scheme and sacrificing in-group members. We performed a mediation analysis in MPlus8 (Muthén & Muthén, 2017) using maximum likelihood estimation with 5,000 bootstrap samples. National narcissism was entered as the predictor variable, group reputation concern as the mediator, and support for opting out of the EU scheme and sacrifice of in-group members as dependent variables (Figure 1). National identification, political ideology, and satisfaction with Johnson were entered as covariates.^4^

We first examined the path from national narcissism to support for opting out of the EU scheme. After accounting for group reputation concern, the direct effect of national narcissism on support for opting out became nonsignificant (β = .09, *b* = .13 [−0.09, 0.35], *p* = .252). The indirect effect of national narcissism on support for opting out of the EU scheme via group reputation concern was significant and positive (β = .09, *b* = 0.14 [0.02, 0.25], *SE* = 0.06, *p* = .020).

We next examined the path from national narcissism to sacrifice of in-group members. The direct effect of national narcissism was significant and positive (β = .28, *b* = .36 [0.13, 0.60], *p* = .002). The indirect effect of national narcissism on in-group sacrifice via group reputation concern was also significant and positive (β = .11, *b* = 0.14 [0.02, 0.26], *SE* = 0.06, *p* = .018).

### Discussion

In Study 1, we showed that national narcissism is related to support for in-group harming actions amid a national crisis. Furthermore, we found evidence suggesting that this relationship is mediated by group reputation concerns. Although satisfaction with Johnson was unsurprisingly associated with support for his decision, the effects of national narcissism remained medium to strong after we adjusted for it in the models. Given the competitive context of U.K.–EU relations, in Studies 2 and 3, we sought to replicate these findings in other intergroup settings to examine their generalizability.

## Study 2: Reduced Testing as Image Management

In Study 2, we tested our hypotheses in a different context, namely, attitude toward testing for COVID-19 in the United States. Testing for COVID-19 is one of the most effective strategies to curb the spread of the virus (Centers for Disease Control and Prevention [CDC], 2020). Yet, testing became a bone of contention in the United States early on in the pandemic, with President Trump expressing his opposition to a large-scale testing program (Segers, 2020). Trump’s argument was that a high number of tests would inevitably result in more reported cases, leading to a poor comparison with other countries. We tested a pre-registered hypothesis that national narcissism would be associated with support for slowing down testing. Our pre-registration included the study design, planned sample size, exclusion criteria, and planned primary analyses.

The defensiveness accompanying national narcissism may increase when an unfavorable comparison with other countries is made salient (Golec de Zavala et al., 2016). In the context of COVID-19, this may be associated with a preference for policies that reinforce an idealistic national image, like downplaying or concealing infection rates, despite the obvious risk this brings to fellow citizens. Thus, in Study 2, we expand on Study 1 by testing the pre-registered hypothesis that inducing an out-group comparison with China’s relative success in combatting the virus (vs. no mention of China) would strengthen this relationship. We also adjust for satisfaction with Trump’s presidency due to his outspoken skepticism toward COVID-19 testing (Segers, 2020). We further checked whether group reputation concern would mediate the relationship between national narcissism and negative testing attitudes (this hypothesis was not pre-registered).

### Method

#### Participants and procedure

We determined sample size a-priori based on a G*power analysis for an interaction effect (between national narcissism and experimental conditions). In Study 1, we observed large correlations, around .50 (*f*^2^ =.33), between national narcissism and image concerns. Because Study 1 was conducted in the context of U.K.–EU relations, we expected a similar effect size in the intergroup comparison condition, yielding a sample of 26 people necessary to obtain 80% power to replicate the effect, assuming α = .05, two-tailed. We expected the effect to be weaker in the no-comparison condition. In such cases, where a 50% attenuation is expected rather than a “knock-out” effect, Giner-Sorolla (2018) recommends using a cell *n* seven times that of the original effect (here, from Study 1). This yields a multiplication of 26 × 14 participants, resulting in a required sample size of 364.

We recruited 399 American Prolific workers on August 7, 2020. As pre-registered, 18 participants were excluded for failing attention checks (seven in the comparison condition, 11 in the no-comparison condition), leaving 381 for further analysis (49.87% women, *M*_age_ = 33.67, *SD* = 11.67, age range 18–74). A majority of participants were White (68.50%), followed by Black or African Americans (13.12%), Asian Americans (7.35%), and Hispanic or Latino Americans (5.00%; see the Supplement for a full breakdown of ethnicity). Most held a university degree (65.62%) and supported the Democratic Party (49.47%; with 28.95% supporting the Republican Party, the rest supporting other parties or not voting).

Participants first filled out measures of national narcissism and identification. Afterward, they were randomly assigned to read one of two ostensible online news articles entitled “Debate on US testing program continues” (see online materials on OSF). In the no-comparison condition, the article outlined the benefits of extensive COVID-19 testing, such as its importance for contact tracing. Skeptical voices were also addressed, such as that more tests will inevitably lead to more cases being reported. In the intergroup comparison condition, participants read the same passage but with an additional paragraph on China’s success in combating the spread of the virus. Participants learned that while Americans’ daily lives were still heavily affected by the pandemic, China had mostly opened up again. Moreover, participants learned that while the United States reported around 60,000 cases on August 1, 2020, China reported only 48. This condition was designed to elicit an unfavorable comparison to a prominent adversary. After having read the passage, participants proceeded to report their attitudes toward testing, and completed other measures related to COVID-19 polices and politics.

### Measures

#### National Narcissism

National Narcissism was measured with the five-item Collective Narcissism Scale (Golec de Zavala, Cichocka, & Bilewicz, 2013), α = .95, *M =* 3.46, *SD =* 1.92 (e.g., “I will never be satisfied until the United States gets all it deserves”).

#### National Identification

National Identification was measured with the single-item social identification measure (Postmes et al., 2013): “I identify with being American” (*M* = 5.99, *SD* = 1.45).

##### Group reputation concern

Participants indicated their agreement with a single item: “The U.S.’s reputation in the world would be damaged if the COVID-19 economic fallout causes China’s economy to exceed that of the United States” (*M* = 4.43, *SD* = 1.74).

#### National Testing Attitudes

National Testing Attitudes were measured with five items reflecting support for more or less testing, secrecy with case numbers, and willingness to test less to protect America’s image, for example: “I would support conducting less testing if that could make the United States look like it is handling the pandemic better.,” or “I would support conducting less testing even though it might hurt Americans in the long run.” This scale, therefore, entails sacrifice of in-group members along with general testing negativity. Two of the items were measured on a 6-point scale and three on a 7-point scale. We produced *Z*-scores for each item and then used their mean to form a global score of testing negativity (α = .82, *M* = −0.03, *SD =* 0.75).

#### Satisfaction with Trump’s Presidency

Satisfaction with Trump’s Presidency was measured on a scale from 0 = *very dissatisfied* to 10 = *very satisfied* (*M* = 3.34, *SD* = 3.44).

### Results

Correlations for variables are presented in Table 3. National narcissism correlated positively with negative testing attitudes, and group reputation concern.

**Table 3.** Zero-Order Correlations Among Study Variables.

Variable	1	2	3	4
1. National narcissism	—			
2. National identification	.40***	—		
3. Negative testing attitudes	.55***	.22***	—	
4. Satisfaction with Trump	.71***	.33***	.62***	—
5. Group reputation concern	.32***	.13*	.31***	.29***

* *p* < .05. **p < .01. ***p < .001.

We used multiple regression analyses to test our hypotheses (Table 4).^5^ Predictors were mean-centered. Experimental condition was effect coded as 1 = comparison and −1 = no comparison. In Step 1, national narcissism was entered as a predictor, and national identification, satisfaction with Trump’s presidency, and condition as adjustment variables. National narcissism was a significant predictor of negative testing attitudes (β = .25, *p* < .001) and so was satisfaction with Trump (β = .46, *p* < .001). The effect of the condition was nonsignificant (β = −.08, *p* = .052). In Step 2, we included the interaction between national narcissism and experimental conditions. The interaction term was significant (β = −.09, *p* = .022, Δ*R*^2^ = .01). However, when we decomposed the interaction it turned out that the effect of national narcissism on testing negativity was positive and significant in both conditions, but contrary to our predictions, it was in fact stronger in the no-comparison condition (β = .36, *b* = 0.14, 95% CI [0.09, 0.20], *p* < .001), than in the intergroup comparison condition (β = .18, *b* = 0.07, 95% CI [0.02, 0.12], *p* = .009). It should be noted that overlapping confidence intervals suggest that this difference is not reliable.

**Table 4.** Regression Analyses of Negative Testing Attitudes.

Predictor	Step 1			Step 2		
	*b*	95% CI	*β*	*b*	95% CI	β
National narcissism	0.10***	[0.06, 0.15]	.25	0.11***	[0.06, 0.15]	.27
National identification	−0.01	[−0.06, 0.03]	−.03	−0.02	[−0.06, 0.03]	−.03
Satisfaction with Trump	0.10***	[0.08, 0.12]	.46	0.10***	[0.08, 0.12]	.45
Condition	−.06	[−0.12, 0.001]	−.08	−0.06*	[−0.12, −0.001]	−.08
National Narcissism **×** Condition				−0.04*	[−0.07, −0.01]	−.09
*F* (*df*)	66.86 (4, 376)***			55.16(5, 375)***		
*R* ^2^	.42			.42		

*Note.* CI = confidence interval.

* *p* < .05. **p < .01. ***p < .001.

#### Group reputation concern as a mediator of negative testing attitudes

We hypothesized that group reputation concern would mediate the relationship between national narcissism and negative testing attitudes. We tested a mediation model in PROCESS 3.5, with 5,000 bootstraps and 95% confidence intervals. National narcissism was specified as the predictor and group reputation was the mediator, and we included national identification, satisfaction with President Trump and experimental condition as covariates.^6^ National narcissism predicted group reputation concern (β = .22, *b* = 0.20, 95% CI [0.07, 0.33], *p* = .002). Furthermore, group reputation concern predicted support for negative testing attitudes (β = .13, *b* = 0.06, 95% CI [0.02, 0.09], *p* < .001). The direct effect of national narcissism on negative testing attitudes was significant (β = .23, *b* = 0.09, 95% CI [−0.04, 0.13], *p* < .001). The indirect effect of national narcissism on negative testing attitudes via group reputation concern was also significant and positive (β = .03, *b* = 0.01, 95% CI [0.002, 0.02], *SE* = 0.01).

### Discussion

We found support for the hypothesis that national narcissism is positively related to testing negativity. This finding lends further support for our notion that national narcissism is associated with greater concern for the in-group image than citizen well-being. Concerns about the in-group’s reputation partially accounted for this effect. This suggests that people high in national narcissism might favor reducing COVID-19 testing as a strategy to bolster a glorious national image. We further found that the effect of national narcissism on testing negativity occurred independently of intergroup comparisons. However, it was slightly weaker in the out-group comparison condition, than in the no-comparison condition. One reason behind this surprising pattern may be that in the comparison condition, testing was not explicitly mentioned as a pandemic response in China (it was only stated in a general sense that “China had made significant progress”). Depending on the participants’ reading, more testing could in fact help compete with China, countering the idea that less testing would make the United States “look better.” In Study 3, we accounted for this possibility and clarified in the outgroup-comparison condition exactly which specific strategy could help promote the in-group image.

## Study 3: Rushing the Vaccine

In Study 3, we sought to replicate the findings from Studies 1 and 2 by testing whether national narcissism predicts support for actions that may endanger in-group members. Here, we focused on controversies about a premature release of a vaccine for COVID-19 that occurred in late summer of 2020. As scientific understanding of COVID-19 increased and vaccine developers made progress, calls were made for an early release of the vaccine (see Lovelace & Higgins-Dunn, 2020). Some advocated for skipping additional vaccine safety tests to expedite the vaccine roll-out. This posed a dilemma. An early release could help curtailing the pandemic more quickly, be seen as a great scientific achievement, and an opportunity to spite adversaries and competitors. Others advocated for caution as a premature vaccine approval jeopardizes people’s health and safety.

We pre-registered the hypothesis that national narcissism will positively predict support for rushing the release of a vaccine for COVID-19. Like in Study 2, we sought to induce out-group comparison (vs. no comparison) by utilizing news of the Russian government releasing its vaccine ahead of others (“Coronavirus,” 2020). We again pre-registered the hypothesis that the positive relationship between national narcissism and support for rushing to a vaccine will be stronger in the comparison condition than the no-comparison condition. Although the results of Study 2 did not confirm this prediction, we suspected that it could have been due to the somehow ambiguous nature of comparisons we used. Thus, in Study 3, we tested our prediction making the dimension of competition clearer (releasing a pre-mature vaccine like Russia). Finally, we sought to conceptually replicate the findings of Studies 1 and 2 that group reputation concern mediates the relationship between national narcissism and support for harmful policies (included as exploratory in our pre-registration).

Vaccinations are a politically sensitive subject, as widespread conspiratorial beliefs are associated with them (Jolley & Douglas, 2014). There is also evidence suggesting that national narcissism predicts general skepticism toward vaccines (Cislak, Marchlewska, et al., 2021). Thus, we accounted for people’s general attitudes toward vaccines in our analyses.

### Method

#### Participants and procedure

We based our sample size on the same power analysis that we used in Study 2 (for the interaction effect between national narcissism and experimental condition). A total of 401 American Prolific workers took part in an online survey on September 28, 2020. As pre-registered, 30 participants were excluded for failing an attention check (17 in the no-comparison condition and 13 in the comparison condition), and one participant was removed for reporting to be under the age of 18, leaving 370 for further analyses (49.46% women, *M*_age_ = 32.58, *SD* = 11.65, age range 18–75). Most had a university degree (59.89%) and supported the Democratic Party (57.84%; 21.08% Republican; the rest voting for another party or not voting). In terms of ethnicity, 67.84% were White, 12.43% were Asian, 10.27% were Hispanic or Latino American, and 5.41% were Black or African American (see the Supplement for more details).

Participants first completed the measures of national narcissism and identification (counterbalanced; see the Supplement). Then, they were randomly allocated to read one of two passages about the COVID-19 pandemic designed to resemble online newspaper articles (see online materials on OSF). In the no-comparison condition, participants read about the ongoing U.S. vaccine development. The article explained that early trials of the American vaccines showed promising results in terms of the vaccine’s effectiveness and safety. However, the crucial “Phase 3,” where the vaccine will be evaluated more rigorously, could take many more months. Participants read that some people believed that America did not have that time: The vaccine should be released as soon as possible, even before the conclusion of “Phase 3” and despite the risk associated. This passage was designed not to elicit out-group comparison. In the comparison condition, participants read the same text as described above but with additional information on Russia’s accelerated vaccine development. They read that Russia planned to release the vaccine in October that year, months ahead of Americans, and that the country’s vaccine had been labeled “Sputnik-V” as a reference to Soviet success in the space race. Participants then proceeded to report their support for rushing the vaccine development (the dependent variable). Finally, participants answered questions on group reputation concern, satisfaction with President Trump, and other questions on COVID-19 policies and politics.

It is important to note that data collection took place months before the actual rollout of any COVID-19 vaccine and before any of the currently approved vaccines completed Phase 3 of clinical trials. Thus, at the time, releasing the vaccines could have been pre-mature. The scenario presented to participants (dilemma about releasing the vaccine before concluding scientific trials) was purely theoretical and had no base in reality. At the end of the study, participants were thoroughly debriefed and directed to official information on the vaccine development.

### Measures

#### National Narcissism

National Narcissism was measured as in Study 2 (α = .90, *M =* 2.74, *SD =* 1.38).

#### National Identification

National Identification was measured as in Study 2 (*M* = 5.73, *SD* = 1.45).

##### Rushing the vaccine development

Participants reported their agreement with three items: “The United States should do everything in its power to be the first country to have a COVID-19 vaccine,” “The U.S. COVID-19 vaccine should be released to the public, even if the vaccine’s efficiency and safety have not been sufficiently demonstrated,” and “The United States should start mass COVID-19 vaccination, even if it might end up endangering the health of some Americans.” We used the mean of these three items to produce a single variable capturing attitudes toward rushing the development of the COVID-19 vaccine (α = .77, *M* = 2.36, *SD* = 1.22).

#### Satisfaction with President Trump

Satisfaction with President Trump was measured on a scale from 0 = *very dissatisfied* to 10 = *very satisfied* (*M* = 2.57, *SD* = 3.32).

#### Group Reputation Concern

Group Reputation Concern was measured with the item “The U.S.’s reputation in the world would be damaged if other countries were quicker in developing and administering a COVID-19 vaccine” (*M* = 2.77, *SD* = 1.56).

#### General Vaccine Support

General Vaccine Support was measured with two items: “In general, I think vaccines are safe and effective” and “In general, I am in favor of vaccination” (*r* = .86, *M* = 6.02, *SD* = 1.17).

### Results

Zero-order correlations are shown in Table 5. In line with our expectations, national narcissism correlated significantly and positively with support for rushing the vaccine development but negatively with general vaccine support.

**Table 5.** Zero-Order Correlations Among Study Variables.

Variable	1	2	3	4	5
1. National narcissism	—				
2. National identification	.44***	—			
3. Support for rushing vaccine	.49***	.18***	—		
4. Satisfaction with Trump	.60***	.34***	.41***	—	
5. Group reputation concern	.40***	.16**	.49***	.30***	—
6. General vaccine support	−.30***	−.08	−.04	−.37***	−.04

* *p* < .05. **p < .01. ***p < .001.

We predicted that the positive relationship between national narcissism and support for rushing the vaccine development would be stronger in the comparison condition than in the no-comparison condition. National narcissism, national identification, satisfaction with Trump, general vaccine support and condition as predictors of support for rushing the vaccine were entered in Step 1 (see Table 6). Predictors were mean-centered and condition effect coded (−1 = no comparison, 1 = comparison). National narcissism was a significant and positive predictor of rushing the vaccine development, (β = .44, *p* < .001). Satisfaction with Trump (β = .25, *b* = 0.09, *p* < .001) and general vaccine support (β = .18, *p* < .001) were both significant and positive predictors of rushing the vaccine. Condition was a negative predictor (β = −.18, *p* < .001), meaning that support for rushing the vaccine development was higher in the no-comparison condition than in the comparison condition.^7^ The interaction term of national narcissism and condition was entered in Step 2. The interaction term was not significant (β = .06, *p* = .55, Δ*R*^2^ = .001).^8^

**Table 6.** Regression Analyses of Support for a Rushed Vaccine.

Predictor	Step 1			Step 2		
	*b*	95% CI	β	*b*	95% CI	β
National narcissism	0.39***	[0.29, 0.49]	.44	0.39***	[0.29, 0.49]	.44
National identification	−0.08	[−0.16, 0.01]	−.09	−0.08	[−0.16, 0.004]	−.09
Satisfaction with Trump	0.09***	[0.05, 0.13]	.25	0.09***	[0.05, 0.13]	.25
General vaccine support	0.19***	[0.09, 0.29]	.18	0.19***	[0.09, 0.28]	.18
Condition	−0.18***	[−0.28, −0.07]	−.15	−0.24*	[−0.48, −0.01]	−.20
National Narcissism **×** Condition				0.02	[−0.05, 0.10]	.06
*F (df)*	33.32 (5, 364)***			27.80 (6, 363)***		
*R* ^2^	.31			.32		

*Note.* CI = confidence interval.

* *p* < .05. **p < .01. ***p < .001.

### Group Reputation Concern as a Mediator of the Relationship Between National Narcissism and Support for Rushing Vaccine Development

We hypothesized that group reputation concern would mediate the relationship between national narcissism and support for rushing a vaccine for COVID-19. We tested a mediation model in PROCESS 3.5, with 5,000 bootstraps and 95% confidence intervals. National narcissism was specified as the predictor and group reputation concern was the mediator, and we entered national identification, satisfaction with President Trump, general support for vaccinations, and experimental condition as covariates.^9^ National narcissism predicted group reputation concern (β = .37, *b* = 0.41, 95% CI [0.27, 0.55], *p* < .001). Furthermore, group reputation concern predicted support for rushing the vaccine (β = .32, *b* = 0.25, 95% CI [0.19, 0.32], *p* < .001). The direct effect of national narcissism was significant (β = .32, *b* = 0.28, 95% CI [0.19, 0.38], *p* < .001). The indirect effect of national narcissism on support for rushing to a vaccine via group reputation concern was significant (β = .12, *b* = 0.10, 95% CI [0.06, 0.15], *SE* = 0.03).

### Discussion

In line with our hypothesis, we found that national narcissism predicted support for an early release of a vaccine for COVID-19 regardless of its risks for citizens’ health and safety. This association was independent of intergroup comparisons—our experimental manipulation did not moderate the effects. These results emerged over and above participants’ general opinions of vaccination. In fact, in line with previous research (Cislak, Marchlewska, et al., 2021), national narcissism was associated with vaccine skepticism. Reputational concerns can help explain this seemingly conflicting finding. Those high in national narcissism would generally be skeptical or even conspiratorial about vaccinations, but the world’s first COVID-19 vaccine would have been something for the nation to boast about and spite rivals. Therefore, national narcissism may differentially fuel pro- or anti-science attitudes depending on how they make the in-group look in the eyes of others (see also Cislak, Cichocka, et al., 2021 for similar findings in the environmental context).

## General Discussion

We present evidence that national narcissism is linked to readiness to sacrifice compatriots to maintain a positive in-group image of the nation on the world stage. Our studies tested these associations in a context where the prioritization of national image can have deadly consequences. In Study 1, we demonstrated that British national narcissism was associated with support for the decision to opt out of a beneficial EU scheme to procure medical equipment. National narcissism also positively predicted the sentiment that opting out of the scheme is the right decision even though it may harm British people. In Study 2, American national narcissism was related to negativity toward expansive testing for COVID-19, which could highlight unfavorable case numbers for the United States. In Study 3, American narcissism was related to support for rushing to release a vaccine for COVID-19 without adequate scientific testing. In all studies, these relationships were mediated by concerns about the in-group’s reputation. They were also independent of whether out-group comparisons were made salient. This adds to a growing literature suggesting that those high in collective narcissism lack concern for their in-group members (e.g., Cichocka et al., 2021).

### National Narcissism and Self-Defeating Image Management

Our findings have important theoretical implications for understanding the potential risks associated with overinvestment in the in-group’s image. Here, a grandiose yet defensive national identity, namely national narcissism, was related to a preference for harmful, even sacrificial, policies aimed at image management. Previous studies have alluded to this in the case of environmental protection (Cislak, Cichocka, et al., 2021; Cislak et al., 2018), support for vaccination policies (Cislak, Marchlewska, et al., 2021), and international cooperation (Marchlewska et al., 2018). We extend these findings by directly tapping into the renunciation of in-group members’ well-being. In essence, collective narcissists’ low regard for in-group members can translate into support for counterproductive policies aimed to save the group’s face.

Our research adds to a growing literature suggesting that those high in collective narcissism lack empathy for their own group members and prioritize their own, personal interests. For example, collective narcissism is associated with the objectification of one’s in-group members (Cichocka et al., 2021) and lower loyalty to the in-group, such as leaving one’s homeland for financial benefits (Marchlewska et al., 2020). Corroborating this view, recent research has linked national narcissism with social cynicism—a negative view on human nature (Marchlewska et al., 2021). Those high in national narcissism may simply be cynical about the fate of their fellow in-group members. Thus, the in-group becomes a tool for self-enhancement, for instance, when deliberately harming the group serves reputation or prestige motives. Of course, such behaviors may be short-sighted and harm the in-group’s reputation in the long run, which might ultimately reflect badly on those scoring high in collective narcissism. In this, collective narcissism resembles individual narcissism, which tends to predict engagement in short-term self-aggrandizing strategies, which harm social relationships in the long term (Cichocka et al., 2021; Morf & Rhodewalt, 2001; Vazire & Funder, 2006). Indeed, our findings have parallels with interpersonal outcomes of grandiose (as compared with vulnerable) narcissism, such as entitlement, grandiose fantasies, exploitativeness, and a disregard of how these behaviors affect others (Miller et al., 2011).

The main effects observed in our studies were at least partially accounted for by concern about the in-group’s reputation. Similarly, past studies linked national narcissism to refusing aid from others due to suspected ulterior or strategic motives of those offering help (Mashuri et al., 2020). Those high in national narcissism, therefore, seem to be willing to refuse benefits to their in-group based on unfounded claims (Cislak et al., 2020). In addition to refusing outside help, hypervigilance to how others perceive the in-group’s status and reputation could prevent those scoring high in national narcissism from taking effective measures domestically. In the context of the COVID-19 pandemic, this meant refusal to cooperate with other countries, opposition to testing extensively, or concluding scientific trials before starting mass vaccination. Those high in national narcissism may be especially prone to follow image-centered leadership promoting such initiatives. For example, national narcissism predicted support for President Trump, who emphasized restoring America’s image and respect in the world (Cichocka & Cislak, 2020; Federico & Golec de Zavala, 2018; Marchlewska et al., 2018).

Overall, in Western politics, national narcissism tends to be associated with political conservatism (e.g., Federico & Golec de Zavala, 2018; Marchlewska et al., 2018). In our studies, national narcissism correlated strongly not only with support for conservative leaders but also with right-wing ideological self-placement (see details in Supplement). National narcissism also correlated with anti-science attitudes in Study 3 (see Cislak, Marchlewska, et al., 2021). Contemporary conservatism and anti-science attitudes may, in part, appeal to those high in national narcissism because they emphasize a return to national glory and independence from others (Cichocka & Cislak, 2020; Sternisko et al., 2021). Although, in the national context, collective narcissism tends to be linked to right-wing beliefs, it should not be interpreted as solely a right-wing phenomenon. The associations between collective narcissism and ideology may depend on the identity context in which collective narcissism is examined. For example, research has demonstrated defensive processes associated with partisan collective narcissism measured among members of both liberal and conservative parties (Bocian et al., 2021; Cichocka et al., 2021).

Importantly, we did not find similar effects for national identification as we did for national narcissism. After accounting for their overlap, national identification was either unrelated or negatively related to support for policies that serve image management. This is consistent with research showing that national identification without the narcissistic component is associated with more desirable intra- and intergroup attitudes (Cichocka et al., 2016; Golec de Zavala, Cichocka, & Iskra-Golec, 2013). For example, after accounting for its overlap with collective narcissism, in-group identification was associated with lower suspicion of out-groups (Cichocka et al., 2016). In-group identification can also have multiple positive consequences for the in-group, such as increased trust, cooperation, and involvement in in-group activities (Bilewicz & Wójcik, 2010; Brewer, 1999), and greater loyalty to the group (Abrams et al., 1998; Ellemers et al., 1997; Marchlewska et al., 2020). Thus, national identification that is confidently held and is less preoccupied with out-group comparison can be related to beneficial outcomes, both in intra- and intergroup relations (Cichocka, 2016).

### Limitations and Future Directions

Certain perceptions of the in-group may give rise to an expectation that individual in-group members endure suffering for the sake of the entire in-group (Kahn et al., 2017). However, national in-groups are large and diverse and have complicated dynamics between their sub-groups. In our research, national narcissism was measured in relation to British or American nationality, but samples were disproportionately White. This brings up important considerations, not addressed empirically in our studies. First, who is deemed a full member of the in-group? Subgroups tend to attribute their own characteristics to the whole in-group (Wenzel et al., 2007). For example, studies suggest that in the United States, White individuals associate the category “American” less with racial or ethnic minorities than themselves (Devos & Banaji, 2005). In turn, they consider themselves the “prototypical” racial group in the United States. Studies further suggest that White individuals’ perceived threat stemming from the decline in their numerical superiority facilitates resistance to growing diversity (Danbold & Huo, 2015). Racial or ethnic minorities may, therefore, be considered to be on the periphery of the national in-group in the mind of the dominant racial or ethnic group.

A subsequent consideration is: Who in the in-group is deemed an “acceptable loss”?^10^ This brings up another limitation of our approach, as our measure of in-group sacrifice assumed equal risk of citizens being harmed by COVID-19. However, there are striking racial disparities in COVID-19 infection risk and disease severity due to social factors (Shah et al., 2020). Many protection measures, such as working from home, are privileges reserved for individuals of higher status. Therefore, an alternative to our proposition that reputation concerns drive sacrificial attitudes is possible. Perhaps, the dominant ethnic group may marginalize ethnic minorities, and then use post hoc rationalizations, such as group-level reputation concerns, to justify discrimination. Future research would do well in applying an intersectional approach in examining specific subgroups within the nation (such as ethnic minorities) as targets of in-group sacrifice perpetrated by the dominant ethnic group (here, White individuals). It is probable that willingness to sacrifice compatriots depends on *who* of *us* is put on the altar.

Some methodological limitations of our studies should be noted. Study 1 was purely correlational, while the experimental manipulation in Studies 2 and 3 yielded somewhat unclear results. To determine whether the main effect observed in Study 1 depended on a competitive intergroup context, we sought to make out-group comparisons salient in Studies 2 and 3. National narcissism did have a positive and significant effect on preference for image management strategies in both conditions; however, the effect did not increase in the comparison conditions. This might suggest that for those high in national narcissism, image concerns might be chronic. Another possibility is that intensive media coverage makes intergroup comparisons constantly salient in the context of the COVID-19 pandemic. Regardless of the intergroup comparison salience, future studies would do well to use longitudinal methods to investigate whether individual changes in collective narcissism over time predict subsequent changes in policy support. It should also be noted that we did rely on single-item measures, most importantly to capture group reputation concern, which may be considered a limitation. These items, however, do show consistent relationships across studies (see the Supplement for more details). Finally, our samples were not representative of their respective countries in terms of ethnicity and political orientation, which limits the generalizability of our results.

## Conclusion

Sometimes people claim to love their nation and yet initiate or support decisions that may severely harm their compatriots. The end goal may be to preserve the in-group’s honor or spite an adversary, but all too often the in-group itself ends up suffering the consequences. Collective narcissists’ obsession with the in-group’s image and craving for recognition of its greatness may mean they forgo opportunities to effectively tackle crises. We found robust evidence that those high in national narcissism preferred image management over protecting fellow citizens in the context of a global pandemic. For people high in collective narcissism, sacrificing in-group members may be a small price to pay to achieve the desired group image.

## Supplemental Material

sj-docx-1-psp-10.1177_01461672221074790 – Supplemental material for A Small Price to Pay: National Narcissism Predicts Readiness to Sacrifice In-Group Members to Defend the In-Group’s Image Click here for additional data file. Supplemental material, sj-docx-1-psp-10.1177_01461672221074790 for A Small Price to Pay: National Narcissism Predicts Readiness to Sacrifice In-Group Members to Defend the In-Group’s Image by Bjarki Gronfeldt, Aleksandra Cislak, Anni Sternisko, Irem Eker and Aleksandra Cichocka in Personality and Social Psychology Bulletin

## Notes

1. Note also that our exploratory mediation models in all studies had sample sizes large enough to detect small-to-medium (β = .26) effect sizes in α and β mediation paths (Fritz & MacKinnon, 2007).

2. We used pairwise exclusion in all regression models. Unless noted otherwise, no multicollinearity was detected.

3. Political ideology was a relatively weak or nonsignificant predictor of the dependent variables in Study 1. For that reason, we did not include it as a predictor in our pre-registered analyses in Studies 2 and 3.

4. Pattern of results remained similar without these covariates.

5. When adjusting for age, gender, and ethnicity, the interaction between national narcissism and experimental condition became marginally significant, β = −.07, *b* = 0.03, 95% CI [−0.06, 0.002], *p* = .068.

6. Although national identification and satisfaction with Trump were not predictive of group reputation concern, experimental condition was (i.e., group reputation concern was higher in the out-group comparison condition, β = .12, *b* = 0.21, 95% CI [0.04, 0.38], *p* = .016).

7. When adjusting for age, gender, and ethnicity, the experimental condition became a nonsignificant predictor, β = −.18, *b* = −0.22, 95% CI [−0.46, 0.02], *p* = .067.

8. In Step 2, we found signs of multicollinearity for condition and the interaction term (tolerance ≈ .20, variance inflation factor ≈ 0.50 for both). Because the interaction was nonsignificant, we refrained from further interpretation and thus this multicollinearity was not an issue.

9. Although national identification and experimental condition did not predict group reputation concern, satisfaction with Trump (β = .13, *b* = 0.06, 95% CI [0.004, 0.12], *p* = .038) and general vaccine support significantly predicted group reputation concern (β = .11, *b* = 0.15, 95% CI [0.01, 0.28], *p* = .031).

10. Our study materials do not allow for a comparison of *who* within the national in-group should be sacrificed (e.g., Black vs. White individuals), but we do include additional analyses with ethnicity as predictors of the dependent variables in Studies 2 and 3, in the Supplement.
