# Supplementary material for: A Small Price to Pay: National Narcissism Predicts Readiness to Sacrifice In-Group Members to Defend the In-Group’s Image
Source: Pers Soc Psychol Bull. 2022 Feb 22;49(4):612–26. doi: 10.1177/01461672221074790 (PMC9989221; doi:10.1177/01461672221074790)
Supplement: sj-docx-1-psp-10.1177_01461672221074790 – Supplemental material for A Small Price to Pay: National Narcissism Predicts Readiness to Sacrifice In-Group Members to Defend the In-Group’s Image [file sj-docx-1-psp-10.1177_01461672221074790.docx]

**Supplementary Materials**

**Study 1 Supplement**

**Ethnicity and Racial Groups**

When broken down by ethnicity and racial groups, Study 1 had 277 White participants, 3 were Mixed, one was Asian or Asian British, one was Black or Black British, nine reported their ethnicity was “other” and seven said they preferred not to say. Therefore, our sample was 93% White. Due to this imbalance, further analysis of ethnicity was not feasible.

**Resistance to the EU**

The survey included various items measuring resistance or scepticism towards the European Union. In addition to the group reputation concern item in main manuscript, “The UK’s reputation in the world would have been damaged by participating in the EU scheme” we also included the following items: “The UK would have looked weak in the eyes of the world had it participated in the EU scheme”; “Refusing to participate in the EU scheme shows the world that the UK doesn’t need the EU”; “Refusing to participate in the scheme sends a clear message to the EU”; “The UK can manage by itself, we don't need the EU's help.” We averaged these items tapping into an index of resistance to the EU. After adjusting for ideology and satisfaction with Boris Johnson, national narcissism predicted resistance to the EU (β=.41, *b*=0.48 [95%CI 0.36, 0.60], *p*<.001), but national identification did not (β=-.04, *b*=-0.03 [95%CI -0.17, 0.08], *p*=.532).

**Ideology**

Right-wing ideology was included in the main analyses for Study 1, so refer to main manuscript for correlations. To examine the effect of ideological extremity, we conducted a square transformation of the original mean centred ideology variable (i.e., quadratic effect). We repeated the analyses in Table 2 by adding ideological extremity as a predictor to examine if the quadratic effect predicted the outcome over and above linear effects of ideology. The effect of ideology remained similar, but ideological extremity did not have an effect on any of the dependent variables (Table S1).

We examined the possible interactions of right-wing ideology and national narcissism (Table S2). There was no significant interaction for support for opting out of the EU scheme (β=.04, *p*=.10). However, there was a significant interaction of right-wing ideology and national narcissism on in-group sacrifice (β=.30, *p*=.006). The relationship was stronger among participants on the right (β=.40, *p*<.001) than on the left (β=.25, *p*=.003). There was also a significant interaction of right-wing ideology and national narcissism on group reputation concern (β=.36, *p*=.002). The relationship was stronger among participants on the right (β=.32, *p*<.001) than on the left (β=.14, *p*=.11).

| **Table S1** | | | |  |  |  |  |  |  |  |  |  |  |  |  |  |
| --- | --- | --- | --- | --- | --- | --- | --- | --- | --- | --- | --- | --- | --- | --- | --- | --- |
| *Regression Analysis of Support for Opting out of the EU Scheme, In-group Sacrifice and Group Reputation Concern, With Ideological Extremism as Additional Predictor* | | | | | | | | | | | | | | | |  |
|  | DV: Opting out of EU scheme | | | | DV: Sacrifice of In-group members | | | | | | DV: Group Reputation Concern | | | | | |
|  | *b* | 95%CI | β | | *b* | | 95%CI | | β | | *b* | | 95%CI | | β | |
| National narcissism | 0.29^***^ | [0.13,0.45] | .19 | | 0.38^***^ | | [0.21,0.54] | | .30 | | 0.48^***^ | | [0.32,0.63] | | .38 | |
| National identification | 0.15 | [-0.02,0.32] | .09 | | -0.09 | | [-0.26,0.08] | | -.06 | | -0.15 | | [-0.31,0.02] | | -.11 | |
| Satisfaction with Johnson | 0.28^***^ | [0.21,0.34] | .50 | | 0.13^***^ | | [0.06,0.19] | | .27 | | 0.14^***^ | | [0.08,0.20] | | .30 | |
| Right-wing Ideology | 0.04 | [-0.05,0.14] | .05 | | 0.11^*^ | | [0.02,0.20] | | .14 | | 0.11^*^ | | [0.02,0.19] | | .14 | |
| Ideological Extremism | 0.01 | [-0.01,0.04] | .04 | | 0.01 | | [-0.02,0.04] | | .04 | | 0.01 | | [-0.02,0.03] | | .02 | |
| *F (df)* | 70.37 (5,287)^***^ | | | | 30.87(5,287)^***^ | | | | | | 41.84(5,286)^***^ | | | | | |
| *R^2^* | .55 | | | | .35 | | | | | | .42 | | | | | |
| **p* < .05. ***p* < .01. ****p* < .001. | | | | | | | | | | |  |  |  |  |  |  |

| **Table S2** | | | |  |  |  |  |  |  |  |  |  |  |  |  |  |
| --- | --- | --- | --- | --- | --- | --- | --- | --- | --- | --- | --- | --- | --- | --- | --- | --- |
| *Regression Analysis of Support for Opting out of the EU Scheme, In-group Sacrifice and Group Reputation Concern, Including The Interaction of National Narcissism and Right-wing Ideology* | | | | | | | | | | | | | | | |  |
|  | DV: Opting out of EU scheme | | | | DV: Sacrifice of In-group members | | | | | | DV: Group Reputation Concern | | | | | |
|  | *b* | 95%CI | β | | *b* | | 95%CI | | β | | *b* | | 95%CI | | β | |
| National narcissism | 0.25^**^ | [0.08,0.42] | .17 | | 0.40^***^ | | [0.24,0.56] | | .32 | | 0.29^***^ | | [0.12,0.46] | | .23 | |
| National identification | 0.16 | [-0.01,0.33] | .10 | | -0.13 | | [-0.29,0.03] | | -.09 | | -0.07 | | [-0.24,0.10] | | -.05 | |
| Satisfaction with Johnson | 0.28^***^ | [0.22,0.34] | .50 | | 0.14^***^ | | [0.08,0.20] | | .29 | | 0.12^***^ | | [0.06,0.19] | | .26 | |
| Right-wing Ideology | -0.06 | [-0.22,0.10] | -.07 | | -0.07 | | [-0.22,0.08] | | -.09 | | -0.10 | | [-0.26,0.06] | | -.13 | |
| Right-wing ideology × National narcissism | 0.04 | [-0.01,0.09] | .16 | | 0.07^**^ | | [0.02,0.11] | | .30 | | 0.08^**^ | | [0.03,0.13] | | .36 | |
| *F (df)* | 71.31(5,287)^***^ | | | | 44.48(5,286)^***^ | | | | | | 33.77(5,287)^***^ | | | | | |
| *R^2^* | .55 | | | | .44 | | | | | | .37 | | | | | |
| **p* < .05. ***p* < .01. ****p* < .001. | | | | | | | | | | |  |  |  |  |  |  |

**Study 2 Supplement**

**Ethnicity and Racial Groups**

When broken down by ethnicity and racial groups, 261 of our participants were White, 50 Black or African American, 28 Asian or Asian American, 19 Hispanic or Latino Americans (of any race), 5 were Native Americans and Alaska Natives, 3 were Native Hawaiians and Other Pacific Islanders, 13 chose “Other” and 2 chose “Prefer not to say”. We computed dummy variables to reflect this, but due to small group sizes, we grouped Native Americans and Alaska Natives, Native Hawaiians and Other Pacific Islanders together (*n*=8). Those who selected “Other” and “Prefer not to say” were group together (*n*=8).

We then entered these variables as predictors in the same moderation analysis as reported in Study 2, with negative testing attitudes as the dependent variable and continuous predictors mean-centred (see Table S3, Step 1). White race was the reference category. Compared to White participants, Black or African American participants were more likely to report negative testing attitudes (β=.12, *p*=.004), but there were no other significant effects of racial groups/ethnicity. In Step 2, we examined the interaction of national narcissism and racial groups/ethnicity, for groups with more than 15 participants (that is, Black or African American, Asian or Asian American, Hispanic or Latino Americans). We found no significant interaction effects.

| **Table S3** | | | |
| --- | --- | --- | --- |
| *Regression Analyses with Negative Testing Attitudes, Including Racial Groups/Ethnicities as Predictors.* | | | |
| Step 1 (Main effects of predictors used in main analyses and ethnicity) | | | |
|  | *b* | 95%CI | β |
| National narcissism | 0.09^***^ | [0.05,0.14] | .24 |
| National identification | -0.01 | [-0.06,0.03] | -.03 |
| Satisfaction with President Trump | .10^***^ | [0.08,0.12] | .45 |
| Hispanic^a^ | -0.12 | [-0.39,0.15] | -.04 |
| Black^b^ | 0.26^**^ | [0.08,0.43] | .12 |
| Asian^c^ | -0.13 | [-0.36,0.10] | -.04 |
| Native^d^ | 0.03 | [-0.38,0.44] | .01 |
| Other racial group/ethnicity | 0.04 | [-0.26,0.34] | .01 |
| Condition | -0.07^*^ | [-0.13,-0.01] | -.09 |
| *F (df)* | 31.51(9,371)^***^ | | |
| *R^2^* | .42 | | |
| Step 2 (interactions between national narcissism and racial groups/ethnicity) | | | |
| NN **×** Condition | -0.03^*^ | [-0.07,-0.002] | -0.09 |
| NN **×** Hispanic^a^ | -0.05 | [-0.21,0.11] | -0.03 |
| NN **×** Black^b^ | 0.07 | [-0.02,0.16] | 0.06 |
| NN **×** Asian^c^ | -0.09 | [-0.30,0.12] | -0.05 |
| *F (df)* | 22.57(13,367)^***^ | | |
| *R^2^* | .43 | | |
| **p* < .05. ***p* < .01. ****p* < .001. NN = National narcissism. | | | |
| ^a^ Hispanic or Latino American (vs. White). ^b^ Black or African American (vs. White). ^c^ Asian or Asian American (vs. White). ^d^ Native Americans and Alaska Natives, and Native Hawaiians and Other Pacific Islanders (vs. White). | | | |

**Ideology**

Ideology was not included in the pre-registered analyses in the main manuscript, so we include correlations of ideology and other study variables in Table S4. It exhibited positive strong or moderate correlations with all study variables. Most notably, it correlated with negative testing attitudes.

| **Table S4** | | | | | |
| --- | --- | --- | --- | --- | --- |
| *Zero-order Correlations Among Study 2 Variables, Including Ideology.* | | | | | |
|  | 1 | 2 | 3 | 4 | 5 |
| 1. National narcissism |  |  |  |  |  |
| 2. National identification | .40^***^ |  |  |  |  |
| 3. Negative testing attitudes | .55^***^ | .22^***^ |  |  |  |
| 4. Group reputation concern | .32^***^ | .13^*^ | .31^***^ |  |  |
| 5. Satisfaction with President Trump | .71^***^ | .33^***^ | .62^***^ | .29^***^ |  |
| 6. Right-wing ideology | .42^***^ | .25^***^ | .39^***^ | .18^***^ | .58^***^ |
| **p* < .05. ***p* < .01. ****p* < .001. | | | | | |

We repeated the regression analysis of Study 2, but including ideology and ideological extremity as predictors (same methodology as described in the Study 1 Supplement). Interestingly, when entered along with ideological extremity, ideology was not a significant predictor of negative testing attitudes (β=.09, *p*=.079). Ideological extremity was, however, a significant negative predictor (β=-.13, *p*=.004), indicating that moderate participants had more negative testing attitudes than those on the ideological extremes (Table S5).

| **Table S5** | | | |
| --- | --- | --- | --- |
| *Regression Analyses with Negative Testing Attitudes as the Dependent Variable, Including Ideology Variables as Predictors.* | | | |
|  | *b* | 95%CI | β |
| National narcissism | 0.11^***^ | [0.06,0.15] | .27 |
| National identification | -0.02 | [-0.07,0.02] | -.04 |
| Satisfaction with President Trump | 0.10^***^ | [0.07,0.12] | .44 |
| Right-wing ideology | 0.02 | [-0.003,0.05] | .09 |
| Ideological Extremism | -0.01^**^ | [-0.02,-0.003] | -.13 |
| Condition | -0.05 | [-0.11,0.01] | -.07 |
| Condition × National narcissism | 0.03 | [-0.06,0.003] | .07 |
| *F (df)* | 41.55(7,373)^***^ | | |
| *R^2^* | .44 | | |
| ^*^*p* < .05. ^**^*p* < .01. ^***^*p* < .001. | | | |

Finally, we examined whether there was an interaction between national narcissism and right-wing ideology (Table S6). The interaction was not significant (β=-.21, *p*=.13).

| **Table S6** | | | |
| --- | --- | --- | --- |
| *Regression Analyses with Negative Testing Attitudes as the Dependent Variable, Including The Interaction of National Narcissism and Right-wing Ideology* | | | |
|  | *b* | 95%CI | β |
| National narcissism | 0.15^***^ | [0.08,0.22] | .37 |
| National identification | -0.02 | [-0.07,0.02] | -.05 |
| Satisfaction with President Trump | 0.09^***^ | [0.07,0.12] | .42 |
| Right-wing ideology | 0.05 | [-0.004,0.10] | .18 |
| Condition | -0.06^*^ | [-0.12,-0.003] | -.08 |
| Condition × National narcissism | -0.03^*^ | [-0.06,-0.001] | .08 |
| National narcissism × Right-wing ideology | -0.01 | [-0.02,0.002] | -.21 |
| *F (df)* | 40.02(7,373)^***^ | | |
| *R^2^* | .43 | | |
| ^*^*p* < .05. ^**^*p* < .01. ^***^*p* < .001. | | | |

**Study 3 Supplement**

**Ethnicity and Racial Groups**

When broken down by ethnicity, 251 participants were White, 46 were Asian, 38 were Hispanic or Latino Americans (of any race), 20 were Black or African American, 4 Native Americans and Alaska Natives, nine said “Other” and two preferred not to say. We coded dummy variables in accordance with the categorisation described in Study 2 Supplement. We then conducted a similar regression model as in Study 2 Supplement, where we add racial/ethnicity dummy variables as predictors (Table S7). Compared to White participants, Native Americans and Alaska Natives, and Native Hawaiians and Other Pacific Islanders were more likely to support rushing the vaccine development (β = .09, *p* = .039). No other significant differences were observed.

Again, we checked for interaction effects for national narcissism and racial groups/ethnicities with 15 or more participants. There was no significant interaction effect for Asian participants (β=.04, *p*=.46, Step 2) nor Black or African American participants (β=-.09, *p*=.068, Step 2). However, the interaction was significant for Hispanic or Latino American participants (β=-.13, *p*=.004, Step 2). We then dissected the interaction effect. While the relationship between national narcissism and support for rushing the vaccine was positive and significant among White participants (β=.47, *b* = 0.42, 95%CI [0.31, 0.52], *p* < .001), it was not among Hispanics or Latino American participants (β=.11, *b* = 0.10, 95%CI [-0.15, 0.35], *p* = .44).

| **Table S7** | | | |
| --- | --- | --- | --- |
| *Regression Analyses with Support for Rushing the Vaccine, Including Racial Groups/Ethnicities as Predictors.* | | | |
| Step 1 (Main effects of predictors used in main analyses and racial groups/ethnicity) | | | |
|  | *b* | 95%CI | β |
| National narcissism | 0.39^***^ | [0.29.0.49] | .44 |
| National identification | -0.08 | [-0.16,0.004] | -.09 |
| Satisfaction with President Trump | .09^***^ | [0.05,0.14] | .26 |
| General vaccine support | .21^***^ | [0.11,0.30] | .20 |
| Hispanic^a^ | -0.12 | [-0.47,0.24] | -.03 |
| Black^b^ | 0.19 | [-0.29,0.67] | .03 |
| Asian^c^ | 0.08 | [-0.25,0.40] | .02 |
| Native^d^ | 1.08^*^ | [0.06,2.10] | .09 |
| Other racial group/ethnicity | -0.03 | [-0.65,0.59] | -.004 |
| Condition | -0.18^***^ | [-0.29,-0.08] | -.15 |
| *F (df)* | 17.26(10,359)^***^ | | |
| *R^2^* | .33 | | |
| Step 2 (interactions between national narcissism and racial groups/ethnicity) | | | |
| NN × Condition | 0.00 | [-0.07,0.08] | .00 |
| NN × Hispanic^a^ | -0.38^**^ | [-0.65,-0.12] | -.13 |
| NN × Black^b^ | -0.28 | [-0.59,0.02] | -.09 |
| NN × Asian^c^ | -0.11 | [-0.39,0.18] | -.04 |
| *F (df)* | 13.34(14,355)^***^ | | |
| *R^2^* | .35 | | |
| **p* < .05. ***p* < .01. ****p* < .001. NN = National narcissism. | | | |
| ^a^ Hispanic or Latino American (vs. White). ^b^ Black or African American (vs. White). ^c^ Asian or Asian American (vs. White). ^d^ Native Americans and Alaska Natives, and Native Hawaiians and Other Pacific Islanders (vs. White). | | | |

**Ideology**

Correlations of ideology with other Study 3 variables are shown in Table S6. Ideology and support for rushing vaccine development correlated moderately.

| **Table S8** | | | | | |
| --- | --- | --- | --- | --- | --- |
| *Zero-order Correlations Among Study 3 Variables, Including Ideology.* | | | | | |
|  | 1 | 2 | 3 | 4 | 5 |
| 1. National narcissism |  |  |  |  |  |
| 2. National identification | .44^***^ |  |  |  |  |
| 3. Support for rushing the vaccine | .49^***^ | .18^***^ |  |  |  |
| 4. Satisfaction with President Trump | .60^***^ | .34^***^ | .41^***^ |  |  |
| 5. Group reputation concern | .40^***^ | .16^**^ | .49^***^ | .30^***^ |  |
| 6. Right-wing ideology | 59^***^ | .36^***^ | .40^***^ | .75^***^ | .34^***^ |
| **p* < .05. ***p* < .01. ****p* < .001. | | | | | |

We added ideology and ideological extremity as predictors to the same interaction analysis as in Study 3 in the main manuscript. Ideology had a marginally significant positive effect (β=.14, *p*=.053), but ideological extremity did not have an effect (β=.02, *p*=.70).

| **Table S9** | | | |
| --- | --- | --- | --- |
| *Interaction Analysis with Support for Rushing the Vaccine as the Dependent Variable, Including Ideology Variables as Predictors.* | | | |
|  | *b* | 95%CI | β |
| National narcissism | 0.37^***^ | [0.26,0.47] | .41 |
| National identification | -0.08^*^ | [-0.17,-0.001] | -.10 |
| Satisfaction with President Trump | 0.06^*^ | [0.01,0.11] | .16 |
| General vaccine support | 0.20^***^ | [0.10,0.29] | .19 |
| Condition | -0.25^*^ | [-0.48,-0.01] | -.20 |
| Condition × National narcissism | 0.02 | [-0.05,0.10] | .06 |
| Right-wing ideology | 0.06 | [-0.001,0.12] | .14 |
| Ideological Extremism | 0.002 | [-0.01,0.02] | .02 |
| *F (df)* | 21.47(8,361)^***^ | | |
| *R^2^* | .32 | | |
| ^*^*p* < .05. ^**^*p* < .01. ^***^*p* < .001. | | | |

Finally, we examined the possible interaction between national narcissism and right-wing ideology (Table S10). The interaction was significant (β=.10, *p*=.034). The effect was stronger for participants on the right (β=.44, *p*<.001) than on the left (β=.25, *p*=.008).

| **Table S10** | | | |
| --- | --- | --- | --- |
| *Regression Analysis with Support for Rushing the Vaccine as the Dependent Variable, Including the Interaction between National Narcissism and Right-wing Ideology.* | | | |
|  | *b* | 95%CI | β |
| National narcissism | 0.34^***^ | [0.23,0.44] | .38 |
| National identification | -0.07 | [-0.16,0.01] | -.09 |
| Satisfaction with President Trump | 0.06^*^ | [0.01,0.11] | .16 |
| General vaccine support | 0.18^***^ | [0.09,0.28] | .18 |
| Condition | -0.24^*^ | [-0.47,-0.01] | -.20 |
| Condition × National narcissism | 0.02 | [-0.05,0.10] | .06 |
| Right-wing ideology | 0.06 | [-0.002,0.12] | .13 |
| National narcissism × Right-wing ideology | 0.03^*^ | [0.002,0.05] | .10 |
| *F (df)* | 22.28(8,361)^***^ | | |
| *R^2^* | .33 | | |
| ^*^*p* < .05. ^**^*p* < .01. ^***^*p* < .001. | | | |

**Counterbalancing of National Narcissism and Identification Variables**

Participants who completed the national identification scale before the national narcissism scale reported higher levels of national identification (*M*=6.00, *SD*=1.30), than those who completed it after (*M*=5.45, *SD*=1.54). An independent samples t-test suggested that the difference was significant (*t*(382)=3.77, *p* < .001). This did not affect the results of the subsequent regression analyses.

**A Note on Single-Item Measures**

**In-group Sacrifice**

In-group sacrifice was operationalised in each study depending on the situation of the pandemic at each given time. The circumstances in which these studies were conducted were time sensitive, and it was important to be quick while these issues were politically relevant.

In Study 1, in-group sacrifice was measured with a single item. Study 1 was an exploratory study conducted in the turbulent first weeks of the pandemic in early spring of 2021. We relied on a multi-item measures in Study 2 (in which we operationalised in-group sacrifice as negativity towards testing for COVID-19 measured with five items) and Study 3 (in which we operationalised in-group sacrifice as rushing the vaccine development, measured with three items; see online Materials). These measures had a high face validity. Note also that in-group sacrifice and national narcissism had similar correlations across studies (Study 1 *r*=.59, *p*<.001, Study 2 *r*=.55, *p*<.001, Study 3 *r*=.49, *p*<.001).

**Group Reputation Concern**

We captured group reputation concern with face valid single items in all studies. These measures showed similar relationships with other study variables (*r*’s with national narcissism, Study 1 *r*=.53, *p*<.001, Study 2 *r*=.32, *p* < .001, Study 3 *r*=.40, *p*<.001).
